# Data on clinical significance of second trimester inflammatory biomarkers in the amniotic fluid in predicting preterm delivery

**DOI:** 10.1016/j.dib.2016.08.030

**Published:** 2016-08-24

**Authors:** Assaad Kesrouani, Elie Chalhoub, Elie El Rassy, Mirna Germanos, Aline Khazzaka, Jamale Rizkallah, Elie Attieh, Norma Aouad

**Affiliations:** Saint Joseph University, Beirut, Lebanon

**Keywords:** Antenatal, Amniotic fluid, Inflammation, Interleukines, MMP-9, Prediction, Preterm

## Abstract

In this article second trimester amniotic fluid biomarkers are measured for correlation with preterm delivery. One additional milliliter of amniotic fluid is collected during amniocentesis for dosages of IL-6, MMP-9, CRP and glucose levels, along with maternal serum CRP and glucose. MMP-9 and Il-6 levels were measured with the corresponding Human Quantikine^R^ ELISA Kit (R&D systems) according to the instructions provided by the manufacturer. Cut-off values for AF MMP-9 and IL-6 were fixed by the kit sensitivity thresholds.

Data includes ROC curves for glucose (Fig. 1), IL-6 (Fig. 2) and MMP-9 (Fig. 3), aiming to search for sensitivity and specificity in the prediction of premature delivery. Statistical analyses are performed with SPSS v20.0 software. Statistical significance is determined using the Mann–Whitney and one way ANOVA test. The association with preterm delivery is performed using a two proportions test. Correlations are measured using the Pearson׳’s coefficient. A *p* value<0.05 is considered statistically significant. The data is presented in the figures provided. Data relied on a previous publication “Prediction of preterm delivery by second trimester inflammatory biomarkers in the amniotic fluid” (A. Kesrouani, E. Chalhoub, E. El Rassy, M. Germanos, A. Khazzaka, J. Rizkallah, E. Attieh, N. Aouad, 2016) [1].

**Specifications Table**

**Table:** 

Subject area	*Biology*
More specific subject area	*Cytokines in amniotic fluid*
Type of data	*Figures*
How data was acquired	*Laboratory dosage of biomarkers using Quantikine*^*R*^*ELISA Kit (R&D systems)*
Data format	*Analyzed*
Experimental factors	*Amniotic and plasma levels of biomarkers*
Experimental features	*Correlation between biomarker levels at 2*nd *trimesters amniocentesis and preterm delivery*
Data source location	*Hotel-Dieu de France University Hospital, Beirut, Lebanon*
Data accessibility	*The data are within this article*

**Value of the data**

• Amniotic levels of interleukin-6, MMP-9, glucose and CRP could be markers of preterm delivery.
• The data are useful for selecting which of the inflammatory biomarkers in the amniotic fluid could indicate future premature labor.
• The data may provide a diagnostic opportunity for anticipating and preventing premature deliveries.
• Methodology can be used for further assessment of correlation between amniotic biomarkers and preterm delivery.

## 1 Data

**Fig. 1:**
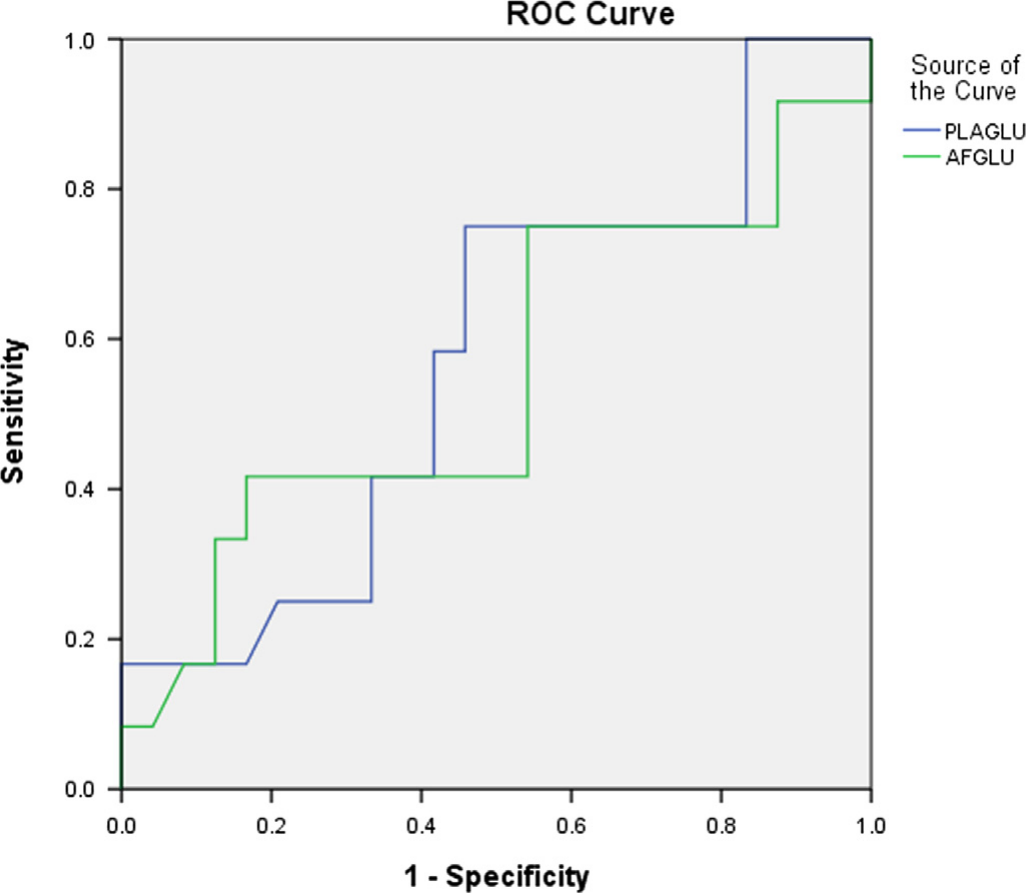
ROC curves for amniotic and plasma glucose levels in predicting preterm labor.

**Fig. 2:**
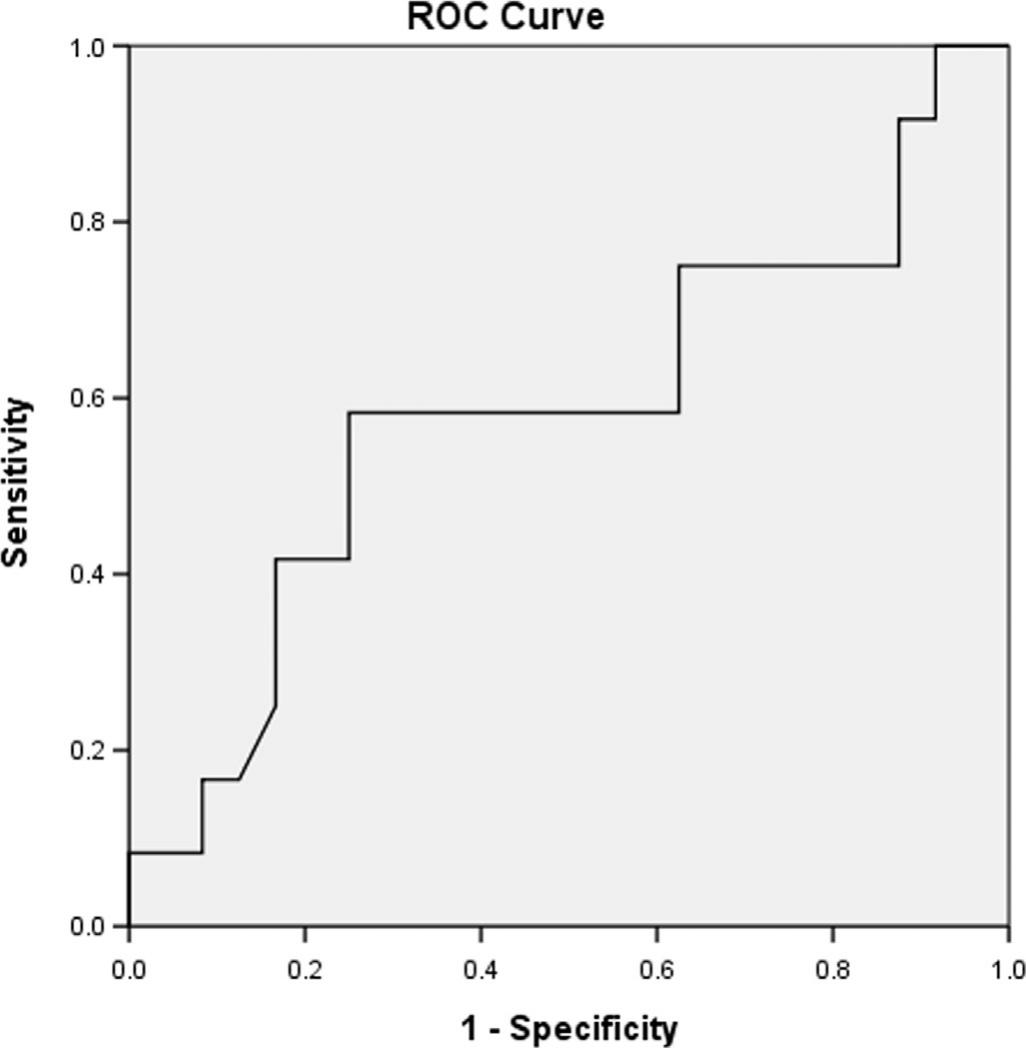
ROC curve for IL-6 levels in predicting preterm labor.

**Fig. 3:**
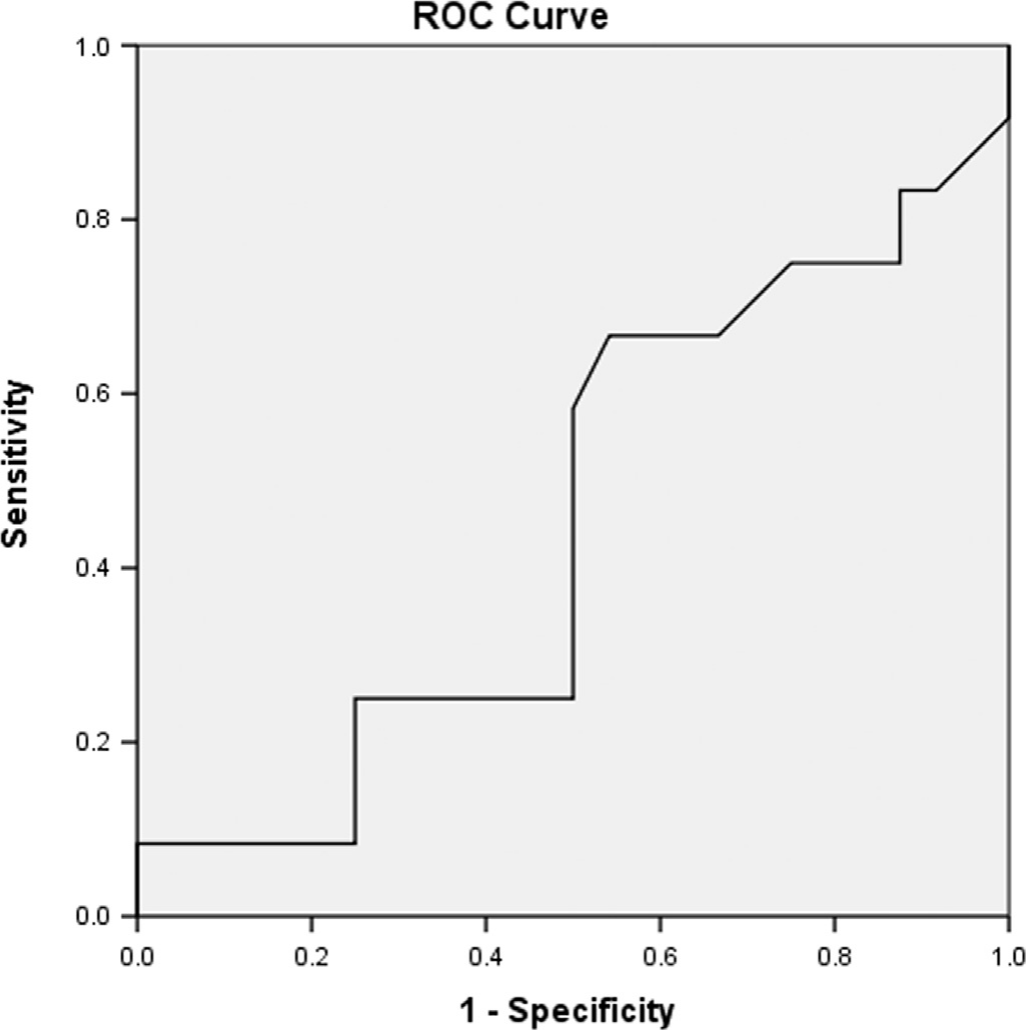
ROC curve for MMP-9 levels in predicting preterm labor.

Amniotic levels of interleukin-6, MMP-9 and CRP are evaluated for risk of preterm delivery. Data includes comparison between levels of term and preterm cases. ROC curves for glucose (plasmatic and intra-amniotic), IL-6 and MMP-9 are presented (Fig. 1, Fig. 2, Fig. 3).

## 2 Experimental design, materials and methods

Data are from of a prospective cohort series at the Obstetrics Department of Hotel Dieu de France University Hospital, Lebanon. It includes patients having a mid-trimester amniocentesis. Indications include advanced maternal age, increased first trimester nuchal translucency, abnormal second trimester biochemical screening test and positive ultrasound soft markers associated with maternal anxiety. Cases with multiple pregnancies, clinical signs of infection or later diagnosis of fetal aneuploidies were excluded. The number of patients enrolled was defined in accordance to the budget allocated by the research council of our institution. This pilot research was approved by the Institutional Review Board of the Faculty of Medicine of Saint Joseph University and eligible patients signed an informed consent for this purpose.

During amniocentesis one additional milliliter of AF was stored for later dosages of IL-6, MMP-9, CRP and glucose levels along with maternal serum CRP and glucose. The data results were not disclosed to both the physician and the patient. MMP-9 and Il-6 levels were measured with the corresponding Human Quantikine^R^ ELISA Kit (R&D systems) according to the instructions provided by the manufacturer. Cut-off values for AF MMP-9 and IL-6 were fixed by the kit sensitivity thresholds and the cutoff was set for AF MMP-9>15 ng/mL and AF IL-6>1430 pg/mL. The cut-off for serum CRP was 9 mg/L and for AF CRP was 0.155 mg/L. No microbiological examination was done because of administrative reasons and because our patients were asymptomatic. All patients were screened for group B streptococcus colonization and bacterial vaginosis with vaginal swab and those who were positive for the test were later excluded. Data aimed to search for possible biomarkers for premature delivery.

Statistical analyses for mean ± standard deviation are performed with SPSS v20.0 software. Statistical significance is determined using the Mann–Whitney and one way ANOVA test. The association between the level of the inflammatory markers and the incidence of preterm delivery is performed using a two proportions test. Correlations are measured using the Pearson׳s coefficient. A *p* value<0.05 is considered statistically significant [1].
