# Supplementary material for: Data on clinical significance of second trimester inflammatory biomarkers in the amniotic fluid in predicting preterm delivery
Source: Data Brief. 2016 Aug 24;9:47–50. doi: 10.1016/j.dib.2016.08.030 (PMC5011160; doi:10.1016/j.dib.2016.08.030)
Supplement: Supplementary file 1 — Supplementary material [file mmc1.docx]

**Conflict of interest**

Regarding the paper paper ‘Data on clinical significance of second trimester inflammatory biomarkers in the amniotic fluid in predicting preterm delivery ‘ submitted to Data in Brief, authors declare no conflict of interest
